# Supplementary material for: Impact of point-of-care ultrasound and routine third trimester ultrasound on undiagnosed breech presentation and perinatal outcomes: An observational multicentre cohort study
Source: PLoS Med. 2023 Apr 6;20(4):e1004192. doi: 10.1371/journal.pmed.1004192 (PMC10079042; doi:10.1371/journal.pmed.1004192)
Supplement: Attachment — Submitted filename: PLOS medicine reviewers Asma 27.12.2022.docx [file pmed.1004192.s002.docx]

27^th^ November 2022

Philippa Dodd MBBS MRCP PhD
Editor
PLOS Medicine

RE: **PMEDICINE-D-22-02818R1
 Impact of Point of Care Ultrasound and routine third trimester ultrasound on undiagnosed
 breech presentation and perinatal outcomes**

Dear Prof Dodd,

Thank you very much for the helpful comments on our manuscript, “Impact of Point of Care Ultrasound and routine third trimester ultrasound on undiagnosed breech presentation and perinatal outcomes”. We have taken these comments into consideration and amended our submission as detailed below. We feel that this helpful review has significantly strengthened this paper and are delighted to resubmit it for your consideration.

Sincerely yours, 

Professor Asma Khalil (on behalf of the authors)

***Requests from the editors:***

*GENERAL*

*Please respond to all editor and reviewer comments detailed below, in full.*

*Please ensure that the study is reported according to the STROBE guideline, and include the completed STROBE checklist as Supporting Information. Please add the following statement, or similar, to the Methods: "This study is reported as per the Strengthening the Reporting of Observational Studies in Epidemiology (STROBE) guideline (S1 Checklist)." The STROBE guideline can be found here: http://www.equator-network.org/reporting-guidelines/strobe/ When completing the checklist, please use section and paragraph numbers, rather than page (or line) numbers.*

Response: The revised version of the manuscript includes the completed STROBE checklist as requested. [Page 7 lines 243-244]

*Throughout, please ensure that all abbreviation are defined at first use*

Response: This has been rectified in the revised version of the manuscript.

*DATA AVAILABILITY STATEMENT*

*PLOS Medicine requires that the de-identified data underlying the specific results in a published article be made available, without restrictions on access, in a public repository or as Supporting Information at the time of article publication, provided it is legal and ethical to do so. Please see the policy at http://journals.plos.org/plosmedicine/s/data-availability and FAQs at http://journals.plos.org/plosmedicine/s/data-availability#loc-faqs-for-data-policy*

*The Data Availability Statement (DAS) requires revision. For each data source used in your study:*

*a) If the data are freely or publicly available, note this and state the location of the data: within the paper, in Supporting Information files, or in a public repository (include the DOI or accession number).*

*b) If the data are owned by a third party but freely available upon request, please note this and state the owner of the data set and contact information for data requests (web or email address). Note that a study author cannot be the contact person for the data.*

*c) If the data are not freely available, please describe briefly the ethical, legal, or contractual restriction that prevents you from sharing it. Please also include an appropriate contact (web or email address) for inquiries (again, this cannot be a study author).*

Response:

We have revised the Data Availability Statement to:

Data cannot be shared publicly because consent was not obtained from women; permission for sharing data was not sought as part of ethical approval. Data is only available following approval from Research Ethics Committee and Confidentiality Advisory Group. Enquiries and requests should be made to the Corresponding author (akhalil@sgul.ac.uk)

*TITLE*

*Please revise your title according to PLOS Medicine's style. Your title must be nondeclarative and not a question. It should begin with main concept if possible. "Effect of" should be used only if causality can be inferred, i.e., for an RCT. Please place the study design ("A randomized controlled trial," "A retrospective study," "A modelling study," etc.) in the subtitle (ie, after a colon).*

Response: The title has been modified to “Impact of Point of Care Ultrasound and routine third trimester ultrasound on undiagnosed breech presentation and perinatal outcomes: a retrospective study“. [Page no-1; line no 1-2]

*ABSTRACT*

*Please structure your abstract using the PLOS Medicine headings (Background, Methods and Findings, Conclusions). Please combine the Methods and Findings sections into one section, “Methods and findings”.*

Response: This has been modified in the revised version of the manuscript. [Page no-2]

** Abstract Background:*

*Please provide the context of why the study is important. The final sentence should clearly state the study question.*

Response: This has been modified in the revised manuscript. [Page no-2; line no 26-27]

** Abstract Methods and Findings:*

*Please ensure that all numbers presented in the abstract are present and identical to numbers presented in the main manuscript text.*

Response: This has been ensured in the revised version of the manuscript.

*Please include the study design, population and setting, number of participants, years during which the study took place, length of follow up, and main outcome measures.*

Response: This has been modified in the revised version of the manuscript. [Page no -2; line no 49-50]

*Please quantify the main results with 95% CIs and p values.*

Response: This has been modified in the revised version of the manuscript. [Page no-2; line no 57-58 and line no 64-65]

*Please include the important dependent variables that are adjusted for in the analyses.*

Response: This is not applicable to our manuscript.

*Please include the actual amounts and/or absolute risk(s) of relevant outcomes (including NNT or NNH where appropriate), not just relative risks or correlation coefficients. (example for absolute risks: PMID: 28399126).*

*Please ensure you clearly report numerators and denominators used to derive percentages*

Response: This has been modified in the revised version of the manuscript. [Page no-2; line no 51-55]

*Please revise your statistical reporting such that all numerical values are clearly defined for the reader. Please define CI, RR etc at first use*

Response: This has been modified throughout the revised version of the manuscript.

*Please include a summary of adverse events if these were assessed in the study.*

Response: Not applicable to this manuscript

*In the last sentence of the Abstract Methods and Findings section, please describe the main limitation(s) of the study's methodology.*

Response: This has been modified in the revised version of the manuscript. [Page no-2; line no 68-70]

** Abstract Conclusions:*

*Please expand the conclusion, in -keeping with the below comments:*

*Please address the study implications without overreaching what can be concluded from the data; the phrase "In this study, we observed ..." may be useful.*

Response: This has been modified in the revised version of the manuscript. [Page no-3; line no 71-75]

*Please interpret the study based on the results presented in the abstract, emphasizing what is new without overstating your conclusions.*

Response: This has been modified in the revised version of the manuscript. [Page no-3; line no 71-75]

*Please avoid vague statements such as "these results have major implications for policy/clinical care".*

Response: This has been modified in the revised version of the manuscript.

*Mention only specific implications substantiated by the results.*

Response: This has been modified in the revised version of the manuscript. [Page no-3; line no 71-75]

*Please avoid assertions of primacy ("We report for the first time....")*

Response: Not applicable.

*AUTHOR SUMMARY*

*At this stage, we ask that you include a short, non-technical Author Summary of your research to make findings accessible to a wide audience that includes both scientists and non-scientists. The Author Summary should immediately follow the Abstract in your revised manuscript. This text is subject to editorial change and should be distinct from the scientific abstract. Please see our author guidelines for more information: https://journals.plos.org/plosmedicine/s/revising-your-manuscript#loc-author-summary*

*Please place this author summary, appropriately structured with bulleted point, under sub-headings (as detailed in our guidance)*

Response: This has been included in the revised version of the manuscript. [Page 3; line no 80-113]

*METHODS and RESULTS*

*Please ensure you have clearly reported the number of participants dates of recruitment, and account for all methods used in your study.*

Response: This has been clearly reported in the methods section.

*Please define "lost to follow-up" as used in this study. Other reasons for exclusion should be defined.*

*Response: The exclusion criteria has been defined. “Lost to follow up” is not applicable to this study.*

*Please define the length of follow up (eg, in mean, SD, and range).*

*Response: Not applicable*

*Please indicate which, if any, factors have been adjusted for in your analyses*

*Response: The analysis plan is explained in the methods section.*

*Did your study have a prospective protocol or analysis plan? Please state this (either way) early in the Methods section.*

*a) If a prospective analysis plan (from your funding proposal, IRB or other ethics committee submission, study protocol, or other planning document written before analyzing the data) was used in designing the study, please include the relevant prospectively written document with your revised manuscript as a Supporting Information file to be published alongside your study, and cite it in the Methods section. A legend for this file should be included at the end of your manuscript.*

*b) If no such document exists, please make sure that the Methods section transparently describes when analyses were planned, and when/why any data-driven changes to analyses took place.*

*c) In either case, changes in the analysis-- including those made in response to peer review comments-- should be identified as such in the Methods section of the paper, with rationale.*

*Response: The analysis plan as described in Methods section was adhered to and there were no data driven deviations. There was no prospective protocol.*

*For all observational studies, we ask that in the manuscript text you clearly indicate:*

*(1) the specific hypotheses you intended to test,*

*(2) the analytical methods by which you planned to test them,*

*(3) the analyses you actually performed, and*

*(4) when reported analyses differ from those that were planned, transparent explanations for differences that affect the reliability of the study's results. If a reported analysis was performed based on an interesting but unanticipated pattern in the data, please be clear that the analysis was data-driven If analyses were performed in response to the peer review process, please clearly indicate this.*

*Response: The analysis plan as described in Methods section was adhered to and there were no data driven deviations.*

*Throughout, we politely request that you be more nuanced in respect to statistical reporting.*

*Please provide the actual numbers of events for the outcomes, not just summary statistics or ORs.*

*Where you report percentages, please clearly report numerators and denominators used to derive them.*

*Please quantify main results with 95% CIs as well as p-values.*

*Where p-values are reported please detail the statistical tests used to determine them.*

Response: We have modified the Methods and Results sections as suggested.

*TABLES*

*Please include appropriate captions which clearly detail the content of the tables, and which define numerical values contained within. Please ensure all abbreviations are also clearly defined for each table e.g., RR, CI, SGHG, NNUH, NE*

*Are any factors adjusted for in the analyses presented here? Please detail such factors in the appropriate caption and where adjusted analyses are presented, please also include the unadjusted analyses to help facilitate transparency of observational data reporting.*

*Please quantify main results reported in the tables with 95% CIs and p-values.*

*Please indicate in the captions the statistical tests used to determine p-values*

Response: We have modified the Tables as suggested.

*Table 2 – suggest removing the second header row between the cohorts – I appreciate that this is to detail the difference in n numbers across the different cohorts but a lot of information is repeated, perhaps unnecessarily, to satisfy that specific requirement. Please revise the presentation of this table to improve accessibility to the reader*

*Please indicate where in the manuscript the tables should be placed*

Response: We have split the table 2 into (3.a and 3.b in the revised version of the manuscript) to improve accessibility and presentation. We have included the other suggestions from the Editor. Table 1 needs to be placed near the first paragraph of the results section followed by Table 2 (near the second paragraph of the results section). Table 3.a and 3.b should be placed near the section on perinatal outcomes in the results section (highlighted in the text).

*FIGURES*

*Please consider using a color palate that is accessible to those with color blindness (i.e., avoids the uses of red and/or green)*

Response: We have modified the Figures as suggested.

*Please indicate where in the manuscript the figures should be placed*

Response: Figure 1 needs to be inserted near the Results, second paragraph, study cohorts section.

*DISCUSSION*

*Please remove all subheadings from the discussion such that it reads as one continuous piece of prose ending in a one paragraph conclusion.*

*Please remove COI statement, funding statement and ethics statement from the end of the main manuscript and include only in the manuscript submission form.*

*Please remove the patient consent statement and incorporate with your ethics statement.*

Response: Discussion section has been formatted according to guidance above and COI, funding, ethics statements have been omitted from the main manuscript.

*REFERENCES*

*For in-text reference callouts, citations should be in square brackets, and preceding punctuation as follows: “…[1,2,3,4].” Please not the absence of spaces where more than one citation is reported.*

*In the reference list, where more than 6 authors contribute to a study please list the first 6 only followed by et al.*

*Please see our website for other reference guidelines https://journals.plos.org/plosmedicine/s/submission-guidelines#loc-references*

*Journal name abbreviations should be those found in the National Center for Biotechnology Information (NCBI) databases.*

Response: References have been edited as per Journal’s requirements as above.

**Comments from the reviewers:**

**Reviewer #1:**

*This study assesses the 'impact' of a universal third trimester ultrasound on breech presentation. It concludes, using 2 'before and after' cohorts from 2 different units introducing 2 different types of 3rd trimester scans, that undiagnosed breech presentation is significantly reduced, that the incidence of breech presentation stays the same, and that the incidence of low Apgar scores is significantly reduced.*

*The first two findings subsequent to a universal scan have been shown before in a single unit cohort previously published in Plos Medicine (salim et al) to which this papers refers, and from which the analysis takes 'informative priors'. The reduction in undiagnosed breech has been shown too, albeit in nulliparous women in research setting, also published in this journal (Wastlund et al) (also cited in this paper). The reduction in Apgar scores was not 'significant' in the Salim study, but with this, it is. That this is so when more data is used should not be considered a surprise: antenatal diagnosis allows more caesarean sections (I note overall numbers not changed but no analysis is specifically presented on cs rates for breech babies- see point below) for breech babies: caesarean sections for breech improve short term outcomes and, according to the term breech trial and huge cohorts from the Netherlands, death.*

*The methodology requires scrutiny by a statistical reviewer and I am not one. This means that I do not fully understand- and apologise for this- how, for instance, despite my point above, the paper concludes that 5 min Apgar scores <7 are reduced, when using a simple chi-squared test amalgamating numbers from Salim et al and from this paper I calculate a chi-square statistic of 2.2251; p= 0.14: i.e. still 'not significant' in lay terms.*

Response: We appreciate the reviewer’s candor and are happy to provide a detailed answer. Bayesian analysis is a statistical estimation method that is especially useful when the outcome of interest is very rare or sampling the target population is hard [1,2]. It uses prior information from already available data (Salim et al) and combines it with the newly collected information (our data) to get combined estimates. Adding raw numbers from two separate studies and performing a chi-squared test is unlikely to work and is also methodologically ill-advised because of the statistical phenomenon called Simpson’s Paradox (<https://en.wikipedia.org/wiki/Simpson%27s_paradox>). The closest method that would resemble a Bayesian analysis would be performing a Mantel-Haenszel RR meta-analysis using data from two studies, but even that is quite different from a Bayesian approach. We believe editors have gathered opinion (Reviewer 4?) from a statistical expert and no objections have been risen about our statistical methods.

1. Quintana M, Viele K, Lewis RJ. Bayesian Analysis: Using Prior Information to Interpret the Results of Clinical Trials. JAMA. 2017 Oct 24;318(16):1605-1606. doi: 10.1001/jama.2017.15574. PMID: 29067406.

2. Laptook AR, et al. Effect of Therapeutic Hypothermia Initiated After 6 Hours of Age on Death or Disability Among Newborns With Hypoxic-Ischemic Encephalopathy: A Randomized Clinical Trial. JAMA. 2017 Oct 24;318(16):1550-1560. doi: 10.1001/jama.2017.14972.

*Although I have outlined that antenatal diagnosis will have benefits, the abstract suggests to the uninitiated reader like me that the paper actually demonstrates a reduction in HIE: 'There was moderate to high probability (posterior probability: 89.5% and 85.1%, respectively) of a reduction of* *hypoxic ischemic encephalopathy (HIE) (RR: 0.23, 95% CI: 0.14-0.38)… '. Yet this is not the case, the numbers simply being inadequate for this: table 2 shows just 2 before and 1 after the scan was introduced. Without fully understanding the methodology this seems disingenuous.*

Response:

Bayesian analysis using informative priors was employed in this study, to counter the fact that number of severe adverse events like HIE, in the context of breech presentation, were quite low, as noted by the reviewer. The “moderate to high probability (posterior probability: 89.5% and 85.1%, respectively) of a reduction of hypoxic ischemic encephalopathy (HIE) (RR: 0.23, 95% CI: 0.14-0.38)… “ is derived using informative priors from Salim et al, and not merely from the cohorts of our study.

*Another issue with this paper is the loss of the 'real world' (what happens in a unit when…) benefits to an impact/ 'changes subsequent to' paper. This is because of there being 2 different units, the intervention was slightly different (proper scan and presentation scan) and ECV was not documented/ discussed. This means it potentially has less relevance for a 'unit' and is more of a 'poor man's' (person's) RCT.*

*I remain surprised at all the research into a universal 'presentation only' scan. I think we know the answer- most breeches become diagnosed. This affects only about 5% of the population. Given the (accepted) benefits of elective caesarean for breech babies have probably been exaggerated (RCOG Greentop Guideline 2017) the overall effect on pregnancy safety is going to be small. And of course performing caesarean sections on all breech babies leads to a loss of skills and therefore probably increased risk for the small (in this study and that of Salim et al) but persistent breeches that still slip through. I make this comment because this pertains to the relevance of this- and other studies such as Salim et al- for overall pregnancy safety; in contrast, a 'proper' scan that could affect the other 95% as well is the one that needs the research attention.*

*I would like to see data on* *how many third trimester scans were actually performed- before and after.*

Response:

We thank the Reviewer for his/her comment, and we agree that finding that an ultrasound scan in the third trimester reduces the incidence of undiagnosed breech presentation is not novel. The focus was on whether this improves perinatal outcomes. If we follow his/her argument that we, as healthcare professionals, would automatically perform a Caesarean delivery, one could argue that an emergency Caesarean section for undiagnosed breech in labour should negate the breech-related neonatal risks. Our finding of an improvement in perinatal outcomes is likely to be important for policy makers and national guidelines. Furthermore, the other novel aspect of our study is the impact of POCUS. Most published studies have focused on the routine third trimester ultrasound. Finally, our study does not address the controversial views of routine implementation of the third trimester ultrasound scan, its components, and benefits as well as cost implications. Therefore, various aspects of implementation are not reported in this study. In fact, we do not have reliable data on how many third trimester scans were actually performed- before and after as this data were not collected prospectively. Moreover, data on the number of third trimester scans performed before and after its implementation have already been reported in other studies (see below). Finally, a model where POCUS is implemented might be an alternative to routine third trimester scan as it is likely to be cheaper and easier to implement in view of the shortage of sonographers.

For example, the study by Wastlund et al [3] on cost effectiveness of third trimester ultrasound derives data from the Pregnancy Outcome Prediction study (POP) [4], where out of 3866 women included in final analysis, 1354/3866 (35%) had selective or clinically indicated ultrasound.

Similarly, in a Swedish study, 5792/24868 (23%) were reported to have a clinically indicated third trimester ultrasound [5].

3.Wastlund D, Moraitis AA, Dacey A, Sovio U, Wilson ECF, Smith GCS. Screening for breech presentation using universal late-pregnancy ultrasonography: A prospective cohort study and cost effectiveness analysis. PLoS Med. 2019 Apr;16(4):e1002778.

4. Gaccioli F, Lager S, Sovio U, Charnock-Jones DS, Smith GCS. The pregnancy outcome prediction (POP) study: Investigating the relationship between serial prenatal ultrasonography, biomarkers, placental phenotype and adverse pregnancy outcomes. Placenta. 2017 Nov;59(Suppl 1):S17–25

5. Bonnevier A, Maršál K, Källén K. Detection and clinical outcome of small-for-gestational-age fetuses in the third trimester—A comparison between routine ultrasound examination and examination on indication. Acta Obstetricia et Gynecologica Scandinavica. 2022;101(1):102–10.

Wilson ECF, Wastlund D, Moraitis AA, Smith GCS. Late Pregnancy Ultrasound to Screen for and Manage Potential Birth Complications in Nulliparous Women: A Cost-Effectiveness and Value of Information Analysis. Value Health. 2021 Apr;24(4):513–21.

*No table data on gestation at birth or mode of birth is presented for the babies who were actually breech.*

Response:

The data requested by the Reviewer have been included in the revised version of the manuscript (Table 2).

*The lack of ECV data is acknowledged as a limitation but inadequately so. The Salim study they quote was not a 'research setting' and its data and discussion of ECV was comprehensive. This lack also causes the problem below:*

*By looking at outcomes in breech babies only at birth (this is not 100% clear at least in the table) an assumption is being made that the universal scan could not alter outcomes for other babies. It could- not just for babies that were breech at birth (eg ECV could be performed which could lead to not being breech and therefore not being included in the outcomes, or some other benefit, or indeed, theoretically, harm). Salim et al 'get around' this by including all babies diagnosed as breech. The method employed by Salim et al has drawbacks too (it did not include those undiagnosed before the universal scan), but the method used here should at least be justified and discussed as a limitation.*

Response:

We agree with the Reviewer that either approach has its own limitations. The data on ECV, whether it was successful or not, and whether the pregnant women were offered or not according to the hospital protocol taking into account the contraindications, and whether the pregnant woman accepted or declined, were not prospectively collected electronically. Therefore, we do not have reliable data to include in the analysis. We have therefore highlighted this point as a limitation in the Discussion section of the revised version of the manuscript.

The pretty Figs 1 and 2 add little useful information bar perhaps demonstrating causality better re undiagnosed breech presentation- but this should be obvious by now.

Response:

We would be happy to remove the figures if this is what the Reviewer is requesting. We would be happy to abide with the Editor’s decision.

In spite of my largely minor reservations, if this stands up to statistical review, this paper cleverly demonstrates one of the benefits to spreading the use of ultrasound in pregnancy.

Response:

We thank the Reviewer for this positive comment.

**Reviewer #2:**

This was a clinically meaningful study, well-written and generally easy to follow. The conclusions are clear and compelling. The role of midwives in delivering POCUS is especially unique and helpful given the potential for this model to be introduced more widely in clinical settings globally. The use of probabilities in the results is confusing to follow, the risk reduction is much easier to understand clinically, however these results are presented in the context of the analysis conducted.

The only suggested changes relate to the interpretation of findings. In the Discussion, I think it is important to clearly mention a) the role of mode of birth in the risk of neonatal adverse outcomes (ie what proportion of women with breech babies proceeded to CS and thus what was the impact of MOB on outcomes, if any, assuming that CS eliminates most of the breech-related risks of vaginal breech birth) and b) the role of parity or the proportion of multiparous women in each group.

Response:

Thank you for the comments. The new table we have added (Table 2) should shed some light on this matter. Elective cesarean sections for breech presentations increased to 76.9% from 60.7%, emergency cesarean sections (30.8% to 17.1%), spontaneous breech (5.5% to 4.4%), and breech extractions deliveries (2.2% to 0.8%) were all decreased (P<0.001). The differences noted in the mode of birth could provide an explanation why the outcomes have improved.

Regarding parity, for the SGH cohort, we had reliable data which shows that the proportion of parous women was similar (49.6% versus 49.2%, p=0.612) in pre and post screening cohorts (Table 1). For the pregnancies with breech presentation at birth (Table 2), the proportion of parous women was also similar (36.2%, versus 37.1%; p=0.612) in pre and post screening cohorts For the NNUH cohort, we did not have reliable electronic data for the pre-POCUS cohort (2015) so the proportions cannot be compared.

Multiparity is a known to be associated with higher chance of spontaneous version from breech to cephalic and vice-versa. In our cohorts, 5/7 (71.4%) and 4/5 (80%) cases of undiagnosed breeches (post implementation of routine third trimester ultrasound and POCUS) in the SGH and NNUH cohorts respectively, were in multiparous women.

**Reviewer #3:**

*1. Line 120-122. "For both centers, pregnancies were grouped according to whether they received routine third-trimester scan (SGH) or POCUS (MNUH)." For the analysis, did the author include all the pregnancies or just include those received routine third-trimester scan at SGH or PROCUS at MNUH?*

Response:

For the analysis, we had two groups (pre and post implementation of either a routine third-trimester scan or POCUS) from the two hospitals. In the post implementation epoch, we included only those pregnancies which received the intervention, according to the hospital.

*2. What's the reason for only including SGH cohort was in Table 1/Figure 1? How about the NNUH cohort?*

Response:

For NNUH, the maternity records were uploaded on electronic database only from April 2015. Therefore, reliable data on demographic parameters like BMI, ethnicity and index of multiple deprivation was not available for the first quarter of 2015. These factors, however, are unlikely to influence the results.

*3. As the log-binomial regression is used to analyze the association between undiagnosed breach presentation at birth and adverse perinatal outcomes, should the outcome be OR (odds ratio) rather than RR (relative risk?)*

Response:

Log-binomial model estimates RR. For rare outcomes, RRs and ORs are similar, therefore may be interchangeably used.

***Reviewer #4:***

*Thanks for allowing me to review your very interesting manuscript. I agree that the data presented show an impressive reduction in the chance of an undiagnosed breech presentation at term after both routine ultrasound screening and POCUS, carried out in two different hospitals. It would, of course, be a more rigorous study design to compare routine antenatal care with both routine ultrasound and POCUS in the same setting, or to have randomised rather than use a before and after design, but based on your data both methods appear to reduce the percentage of breech presentations that are undiagnosed at the start of labour or membrane rupture. I have a few minor points to make which could lead to some minor revisions:*

*1. I found the phrase 'the rate of* *undiagnosed breech' a bit confusing as it suggested to me that, for example, 14.2% of babies were presenting in labour as undiagnosed breeches. It might be better to say the '**percentage of all term breech presentations that were undiagnosed was 14.2%.....'*

Response:

As suggested by the reviewer, we have modified the phrase throughout the revised version of the manuscript.

*2. The statement that there was no significant change in the rate of vaginal breech birth (and the figures are given of 10 per 10,000, which was reducing to 5 per 10,000 after the scanning interventions) is somewhat surprising given the very significant change (reduction) in the percentage of undiagnosed breech presentations. Are you able to explain that?*

Response:

We thank the reviewer for this comment. The significant reduction in the percentage of undiagnosed breech presentation may not be reflected in a similar scale of reduction of breech vaginal deliveries as most of these women would have an emergency caesarean section, even when they are diagnosed with breech presentation for first time in labour (as also pointed by the reviewer in their comment below).

3. *These data would suggest to me that prior to the intervention most breech babies were born by c/section, so it is then a little hard to understand how such an improvement in perinatal outcome has been achieved. I realise that the outcome data were secondary outcomes but the paper seems to argue that there is a high rate of morbidity/mortality associated with vaginal breech birth, yet the improvement in perinatal outcomes has occurred without much change in the rate. Indeed, given the numbers quoted the numbers of term vaginal births at SGH during the study period would have reduced from about 17 in the pre-ultrasound period to 4 after. To have seen a measurable reduction in the rate of low Apgar scores and rates of HIE would be remarkable, and would imply that outcomes for vaginal breeches were very poor in the pre-ultrasound period. Can you comment further on this?*

Response:

The reviewer is asking whether outcomes were so poor for breech deliveries in the pre-screening era that they were improved with time without an appreciable change in delivery method (1% increase in overall elective Caesarean section rate). The new table we have added (Table 2) should shed some light on this matter. Elective cesarean sections for breech presentations increased to 76.9% from 60.7%, emergency cesarean sections (30.8% to 17.1%), spontaneous breech (5.5% to 4.4%), and breech extractions deliveries (2.2% to 0.8%) were all decreased (P<0.001). The differences noted in the mode of birth could provide an explanation why the outcomes have improved.

*3. A minor point is that data is a plural noun and is sometimes used as a singular one (e.g. page 172)*

Response:

We are sorry for the oversight, and have now made corrections throughout the revised version of the manuscript.

4. *Finally, I was left uncertain as to whether or not you would advocate a policy of routine ultrasound or POCUS or a hybrid of the two depending on risk? Or do you have plans to compare the two approaches in the same setting?*

Response:

Based on the findings of our study and previous published literature, there appears to be a definite role of ultrasound for accurate determination of fetal malpresentation, however, due to the design of the study – studying the impact of two different modalities in two different populations – we can not advocate one over the other. As rightly pointed out by the reviewer, the next step would involve head-to-head comparison of the two modalities in the same population along with the cost-effectiveness, which remains crucial before any policy change.
