# Supplementary material for: Impact of point-of-care ultrasound and routine third trimester ultrasound on undiagnosed breech presentation and perinatal outcomes: An observational multicentre cohort study
Source: PLoS Med. 2023 Apr 6;20(4):e1004192. doi: 10.1371/journal.pmed.1004192 (PMC10079042; doi:10.1371/journal.pmed.1004192)
Supplement: Attachment — Submitted filename: PLoS reviewers comments 21.01.2023 Asma.docx [file pmed.1004192.s003.docx]

20^th^ January 2023

Philippa Dodd MBBS MRCP PhD
Editor
PLOS Medicine

RE: **PMEDICINE-D-22-02818R1
 Impact of Point of Care Ultrasound and routine third trimester ultrasound on undiagnosed
 breech presentation and perinatal outcomes**

Dear Prof Dodd,

Thank you very much for the helpful comments on our manuscript, “Impact of Point of Care Ultrasound and routine third trimester ultrasound on undiagnosed breech presentation and perinatal outcomes”. We have taken these comments into consideration and amended our submission as detailed below.

Kind regards, 

Professor Asma Khalil (on behalf of the authors)

*Requests from Editors:*

*GENERAL*

*Thank you for your detailed and considerate responses to previous editor and reviewer comments. Please see below for further comments that we require you respond to in full.*

*TITLE*

*Thank you for revising your title suggest “Impact of Point of Care Ultrasound and routine third trimester ultrasound on undiagnosed breech presentation and perinatal outcomes: an observational multi-centre cohort study” or something similar.*

**Response** – We thank the editors for their suggestion and have modified the title as suggested.

*ABSTRACT*

*Thank you for revising your abstract and for including p-values as well as 95% CIs. For each outcome measure reported we request that both p-values and 95% CIs are reported. If not please clearly state the reasons why not, for purposes of transparent data reporting (please remember that when reporting p-values to report as p<0.001 or where higher the exact p-value as p= 0.002, for example).*

*Suggest reporting as follows – for example, line 59 - (RR: 59 0.29, 95% CI [0.20–0.38], p<0.001) for optimal reader accessibility.*

*Please note the use of parentheses around the CIs and the absence of the semi-colon. You may wish to consider separating upper and lower limits with commas rather than hyphens (as these can be confused with negative values) but we leave it to your discretion.*

*Please check and revise throughout, including in the main manuscript text and tables where relevant – please see below also*

**Response** – In our study, outcome measures have been analysed by Bayesian informative priors which do not yield p values but posterior probabilities and credible intervals. We have reported posterior probabilities and credible intervals in the text as well as the tables. Where available, e.g., Table 1 and 2, p values have been stated in the format as required by the journal.

*AUTHOR SUMMARY*

*Thank you for including an author summary which reads very nicely.*

*Line 100: we appreciate the explanation of your chosen methodological approach, suggest revising to ensure that the statement is interpretable to the more general reader – some may be unfamiliar with the concept of informative priors, for example*

*Line 103: “incidence of percentage…” suggest removing of percentage perhaps?*

*Line 104: suggest quantifying “drastically (a percentage will be adequate here)*

*Line 113: It would be helpful to elaborate on the reasons why assessing cost-effectiveness is necessary – i.e. for feasibility of implementation on a wider scale*

**Response** – Thank you for the suggestions, we have modified the section.

*METHODS and RESULTS*

*Please revise statistical reporting as suggested above*

*please quantify all reported percentages with numerators and denominators, for example line 271, “(12.9 vs 11.5%, p=0.029)” - please check and amend throughout*

*Please also report CIs where p-values are reported, if not please clearly state why not, for purposes of transparent data reporting*

**Response** – We have now quantified all reported percentages with numerators and denominators.

*TABLES*

*Currently journal requirements stipulate that where 95% CIs are presented, including in tables, that p-values are also reported. Please include p-values but if not in your rebuttal letter, please clearly state the reasons why not, for transparency of data reporting*

**Response:** As stated above, Bayesian analysis does not report p values but posterior probabilities and credible intervals. We have reported posterior probabilities and credible intervals in the text as well as the tables. Where available, e.g., Table 1 and 2, p values have been stated in the format as required by the journal.

*Table 3.a – suggest removing the SGH cohort row – perhaps a remnant of the previous version*

**Response**: Apologies for the oversight, the row is now removed.

*DISCUSSION*

*Please remove the sub-heading conclusion.*

**Response** – The sub-heading is removed.

*Line 404: given the observational nature of the study suggest tempering the language here slightly, “Our data suggest that a policy….could be effective in…” or something similar, for example*

**Response** – We have modified the language as suggested.

*SOCIAL MEDIA*

*To help us extend the reach of your research, please provide any Twitter handle(s) that would be appropriate to tag, including your own, your co-authors’, your institution, funder, or lab. Please include these in the manuscript submission form when you re-submit your manuscript*

**Response** – Following Twitter handles could be used - @ProfAsmaKhalil, @kalafaterkan_md, @SmritiPrasad5, @StGeorgesUni

*Comments from Reviewers:*

*Reviewer #1: the authors have addressed all points satisfactorily.*

**Response** – We thank the reviewer for the positive comment.

*Reviewer #2: Thank you for your thorough attempts to rectify requested changes. The manuscript has been satisfactorily updated and improved.*

**Response** – We thank the reviewer for the positive comment.

*Reviewer #3: I appreciate authors' efforts in addressing the questions and revising the paper. I have no further comments.*

**Response** – We thank the reviewer for the positive comment.

*Reviewer #4: Thanks for responding to my previous comments and to those of other reviewers. I have three relatively minor additional points to make about your revised manuscript;*

*1. Line 50 (Abstract). Can you confirm that the statement 'the rate of breech presentation in labour was consistent across all groups (3-4%)' is correct and does this refer to all births? It seems very high if the usual exclusions of preterm, multiples, congenital anomalies are applied.*

**Response** – We thank the reviewer for the comment. The rate of breech presentation (3-4%) is based on the study population – after exclusion of preterm, multiple pregnancies and congenital anomalies.

The incidence of breech presentation at term is quoted as 3-4%^1^, however large population-based studies report a large variation in incidence. Rietberg et al^2^ from the Netherlands have reported that the median percentage of breech presentation at term in their hospital population was 8.2% with a variation among hospitals of 4.2%-12.4%. Therefore, we do not consider the incidence in our study to be unusually high.

1) Fruscalzo A, Londero AP, Salvador S, Bertozzi S, Biasioli A, Della Martina M, et al. New and old predictive factors for breech presentation: our experience in 14 433 singleton pregnancies and a literature review. The Journal of Maternal-Fetal & Neonatal Medicine. 2014 Jan 1;27(2):167–72.

2) Rietberg CCTh, Elferink-Stinkens PM, Brand R, van Loon AJ, Van Hemel OJS, Visser GHA. Term breech presentation in The Netherlands from 1995 to 1999: mortality and morbidity in relation to the mode of delivery of 33,824 infants. BJOG: An International Journal of Obstetrics & Gynaecology. 2003;110(6):604–9.

*2. Line 116 (Introduction). Similarly, is this figure of 3-4% used in the Introduction inclusive of all births, and does it exclude multiple pregnancies?*

**Response** – Similar as above.

*3. Line 175. I would think that 'Southwest London' is usually written as 'South West London' but as I am not a resident of London I am happy to be corrected.*

**Response** – The reviewer is correct, and we have edited the text.
